# Adipocyte lipolysis affects Perilipin 5 and cristae organization at the cardiac lipid droplet-mitochondrial interface

**DOI:** 10.1038/s41598-019-41329-4

**Published:** 2019-03-18

**Authors:** Mita Varghese, Victoria A. Kimler, Fariha R. Ghazi, Gurnoor K. Rathore, Guy A. Perkins, Mark H. Ellisman, James G. Granneman

**Affiliations:** 1 Center for Integrative Metabolic and Endocrine Research, Wayne State University School of Medicine, Detroit, MI 48201 USA; 2 National Center for Microscopy and Imaging Research, University of California, San Diego, La Jolla, CA 92093 USA

## Abstract

This study investigated the effects of elevated fatty acid (FA) supply from adipose tissue on the ultrastructure of cardiac lipid droplets (LDs) and the expression and organization of LD scaffold proteins perilipin-2 (PLIN2) and perilipin-5 (PLIN5). Stimulation of adipocyte lipolysis by fasting (24 h) or β3-adrenergic receptor activation by CL316, 243 (CL) increased cardiac triacylglycerol (TAG) levels and LD size, whereas CL treatment also increased LD number. LDs were tightly associated with mitochondria, which was maintained during LD expansion. Electron tomography (ET) studies revealed continuity of LD and smooth endoplasmic reticulum (SER), suggesting interconnections among LDs. Under fed *ad libitum* conditions, the cristae of mitochondria that apposed LD were mostly organized perpendicularly to the tangent of the LD surface. Fasting significantly reduced, whereas CL treatment greatly increased, the perpendicular alignment of mitochondrial cristae. Fasting and CL treatment strongly upregulated PLIN5 protein and PLIN2 to a lesser extent. Immunofluorescence and immuno-electron microscopy demonstrated strong targeting of PLIN5 to the cardiac LD-mitochondrial interface, but not to the mitochondrial matrix. CL treatment augmented PLIN5 targeting to the LD-mitochondrial interface, whereas PLIN2 was not significantly affected. Together, our results support the concept that the interface between LD and cardiac mitochondria represents an organized and dynamic “metabolic synapse” that is highly responsive to FA trafficking.

## Introduction

Intracellular lipid droplets (LD) are *bona fide* organelles that play critical roles in fatty acid (FA) storage and mobilization. Cytosolic LDs consist of a neutral lipid core, predominantly triacylglycerols (TAG) and steryl esters that are surrounded by a phospholipid monolayer^1,2^. LDs contain one or more member of the perilipin (PLIN) protein family, which serve as scaffolds for assembling protein complexes and activating acylglycerol lipases^3–8^. PLIN1 and PLIN4 are largely limited to adipose tissue, while PLIN2 and PLIN3 are ubiquitously expressed^3,4^. PLIN5 is restricted to tissues that have high levels of mitochondrial FA oxidation, including skeletal muscle, heart and liver^5–8^. PLIN1 and PLIN2 are constitutively associated with LDs and are degraded by proteasomal and/or lysosomal pathways when not bound to LDs^7,9,10^. In contrast, PLIN3, 4 and 5 are termed “exchangeable” PLIN proteins that can traffic between LDs and cytosol^11^. Based on these differences in protein stability, it was proposed that PLIN3, 4, and 5 bind to more transient pools of LDs, whereas PLIN1 and PLIN2 associate with more constitutive pools of LDs^11^.

The hydrolysis of TAG stored in LDs into FAs and glycerol occurs on the LD surface and is a dynamic and highly regulated process. Adipocyte lipolysis is mediated by the stimulation of the β-adrenergic receptors, which triggers adenylyl cyclase to elevate cAMP levels and activate protein kinase A. Cardiomyocytes rely upon lipolysis to provide FAs to mitochondria for β-oxidation to drive the production of ATP^12^. Circulating TAG-rich lipoproteins and albumin-bound FAs released from adipose tissue supply FAs directly to mitochondria for oxidation or esterification to TAG for temporary storage in cytoplasmic LDs^12–16^. Compared to investigations in adipose tissue or liver, data on cardiac LDs is rudimentary^17^. Studies in patients with diabetes and/or obesity have found an association between increased cardiac TAG accumulation and cardiac dysfunction, suggesting that elevated intramyocellular lipid levels and increased FA β-oxidation may exert a toxic effect on the myocardium^12,18^. Therefore, maintaining cardiomyocyte lipid homeostasis appears to be critical for proper cardiac metabolism and function.

In the heart, PLIN5 overexpression has been reported to promote neutral lipid storage as well as mobilization^10,19–21^. PLIN5 expression is upregulated by fasting, a condition that increases FA mobilization from adipose tissue and increases FA utilization in heart, liver and skeletal muscle^10,19,20^. More recent studies have shown that PLIN5 protects the heart from oxidative burden by sequestering FA from excessive oxidation^22^. The precise functional role of PLIN2 in heart is yet to be established. Interestingly, increasing the expression of PLIN2 elevates the TAG content of fibroblasts^23^, HEK 293 cells^24^, hepatic stellate cells^25^, and COS-7 cells^26^, whereas PLIN2-null mice demonstrate reduced hepatic TAG concentrations compared to their wild-type littermates^27^. These observations suggest that PLIN2 may either promote TAG synthesis or reduce TAG lipolysis, as both will lead to increases in TAG content. By analogy to the established role of PLIN1 in regulating adipocyte TAG hydrolysis^21,28–35^, cardiac PLIN proteins PLIN5 and PLIN2 may play a role in determining the metabolic properties and functions of cardiac LDs and might be a key to the balance between excess FA sequestration and oxidation.

Conventional transmission electron microscopy (TEM) studies have found LDs closely apposed to many organelles involved in FA metabolism including the smooth endoplasmic reticulum (SER), mitochondria and peroxisomes^36–39^. Previous studies have reported the close proximity of PLIN5-coated LDs to mitochondria^40,41^. LDs are thought to originate from the SER by triglyceride and steryl esters accumulation between the two SER membrane leaflets^42–47^. However, it is unclear whether the continuity between the SER and the LDs is maintained^48^. Such contacts could play an important role in directing FA flux and thereby limiting toxic effects that might be residual from intracellular ceramide generation. The morphological details of LD associations with mitochondria and SER are unclear because of the difficulty in capturing fine structural details with thin section TEM methods. In recent years however, electron tomography (ET) has overcome certain limitations of conventional TEM methods. ET computes full 3-D reconstructions of thick (0.2–2 μm or greater) sections from tilt-series images obtained with intermediate or high-voltage electron microscopes^49,50^. ET is uniquely equipped to discriminate, at high resolution, the spatial relationship between closely associated membranes and LDs.

The present study investigated the organization of cardiac LDs and cardiac PLIN expression under control conditions and following LD expansion by nutritional and pharmacological treatments that promote the supply of FA from adipose tissue. Analysis of cardiac LDs by high resolution TEM, Fluoronanogold (FNG) labeling, and ET studies suggested the potential for a ‘metabolic synapse’ at the PLIN5-coated LD-mitochondrial interface for effective FA efflux and influx.

## Results

### Augmented adipocyte lipolysis promotes expansion of cardiac LDs

**Figure 1:**
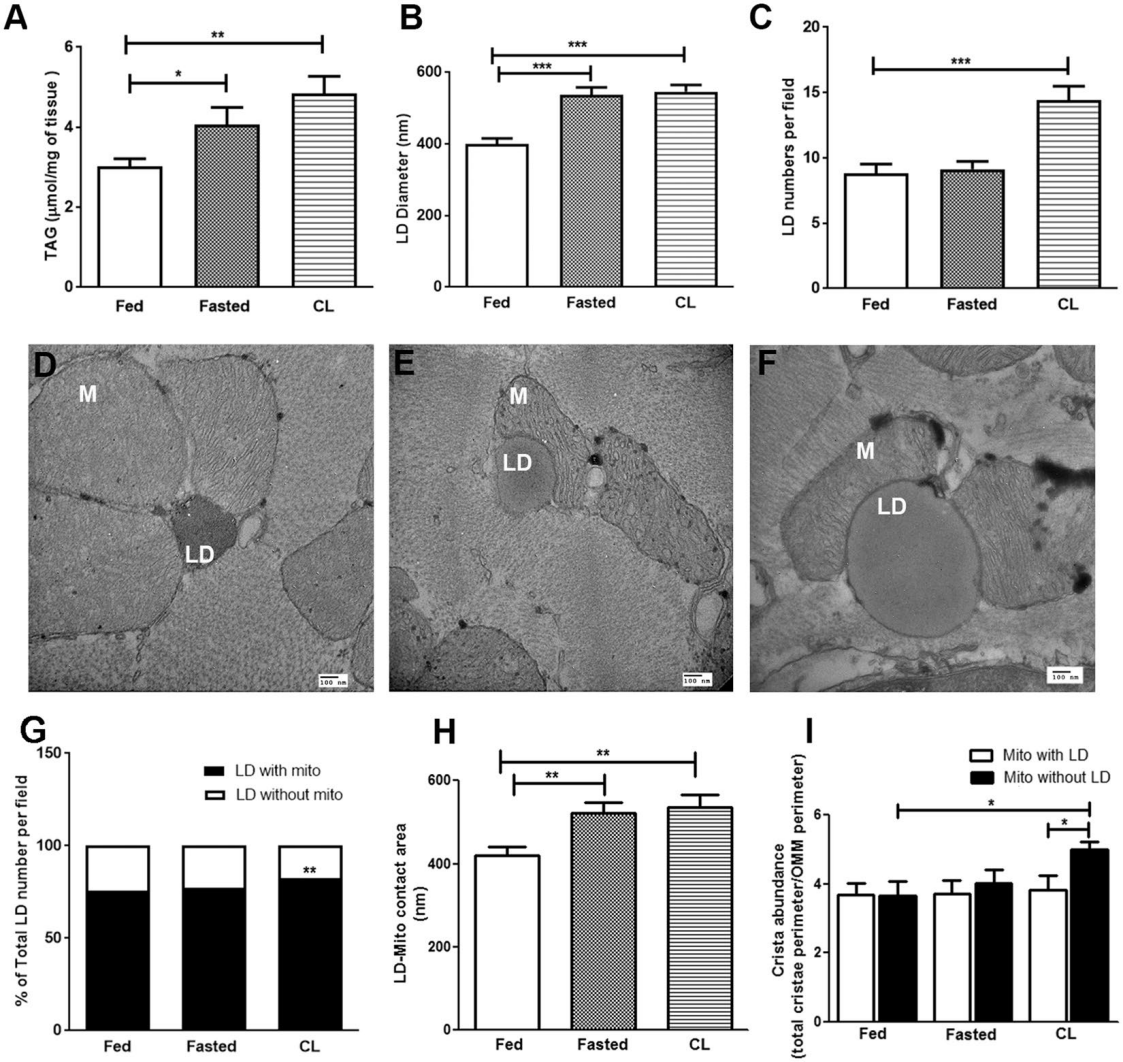
Cardiac LDs and mitochondria respond to nutritional and pharmacological stimuli. (**A**) Triacylglycerol (TAG) levels of cardiac tissues determined in fed, fasted and CL conditions showed a significant increase with fasting (*p < 0.05, t-test, N = 5) and CL treatment (**p < 0.005, N = 5) over control. (**B**) LD size measurements across different conditions shows a significant increase in LD diameter with fasting and CL treatment (***p < 0.0001, t-test). Average LD diameters per field in fed condition = 399.4 ± 15.93 nm, fasting = 536.5 ± 21.45 nm; and CL = 546.2 ± 18.19 nm (N > 100 LDs; N = >5 sections/condition; N = 3–4 mice/condition). (**C**) Graph showing LD numbers in fed, fasted and CL conditions. CL condition showed a significant increase in LD numbers associated with mitochondria (N > 200 LDs; N = >5 sections/condition; N = 3–4 mice/condition, ***p < 0.0001, t-test). TEM micrograph of cardiac tissue from (**D**) Fed condition shows a small LD surrounded by a cluster of mitochondria (M). (**E**) Fasting (24 h) condition showing an increase in the LD size and, (**F**) CL (8 h) condition showed a further increase in LD size compared to fed and fasted heart LD; scale bar = 100 nm. (**G**) Graph showing proportion of LDs in contact with mitochondria in fed, fasted and CL conditions. CL conditions showed a significant increase in LD numbers associated with mitochondria. (**p < 0.05, two-way ANOVA). (**H**) Measurements of the area covered by LD-mitochondria interface showed that LDs in fasted and CL conditions were in contact with one or more mitochondria (**p < 0.005, t-test, 3–4 mice per condition, number of LDs counted >150). (**I**) The cristae abundance determined from the ratio of total cristae/outer mitochondrial membrane (OMM) perimeters showed that the cristae were most abundant in mitochondria from CL condition (*p < 0.05, t-test, N = 10 tomograms per group). In CL conditions, mitochondria without LD showed a significant increase in cristae abundance over those associated with LD (*p < 0.05, t-test). Cristae abundance was also significant over mitochondria without LDs in control conditions (*p < 0.05, t-test).

We examined how cardiac LD content and morphology are affected by physiological and pharmacological mobilization of FAs from adipose tissue. During fasting, reduction in plasma insulin stimulates adipose-tissue lipolysis, which provides the major source of FA for systemic demands. Extended fasting increases TAG levels and intramyocellular LDs in the heart when supply of FA from adipose tissue exceeds oxidation^51–53^. β3-adrenergic receptors are highly expressed in adipocytes^54^ and systemic injection of selective β3-adrenergic receptor agonist CL 316,243 (CL) stimulates massive adipocyte lipolysis, leading to insulin release and sequestration of FA as TAG in numerous peripheral tissues^55,56^. As expected, both fasting and CL treatment significantly increased myocardial TAG levels (Fig. 1A) and FFA levels (Supplementary Fig. S1). TEM of thin section cardiac tissues confirmed that fasting and CL treatment increased LD size (Fig. 1B). We observed a significant increase in LD number following CL treatment, but not after fasting (Fig. 1C). Representative micrographs (Fig. 1D–F) show the increase in LD size in each condition.

LDs are the principal source of FA for mitochondrial beta-oxidation^12^ and it is well known that LDs are closely associated with mitochondria (Fig. 1D–F). To determine whether acute LD expansion affected mitochondrial association with LDs, we analyzed TEM micrographs and measured the proportion of LDs that contacted mitochondria in each condition (Fig. 1G). Under basal conditions, 77 ± 3% of LD contacted mitochondria, and the percentage was not significantly altered by overnight fasting. Acute CL treatment, which generated new LDs, increased the percentage of droplets contacting mitochondria (82 ± 2%, p < 0.05 vs control). Fasting and CL treatment significantly increased LD diameter and the LD area contacted by mitochondria. (Fig. 1H).

Because the great majority of electron transport chain molecules reside on the cristae membranes^57,58^, abundance of cristae suggests increased metabolic activity^59^. We analyzed mitochondria for cristae abundance under various conditions using ET. We did not observe any differences in cristae abundance between mitochondria that were associated or not associated with LD under fed *ad libitum* (fed) or fasting conditions (Fig. 1I). CL treatment also did not affect cristae abundance in mitochondria that contacted LD. Unexpectedly, CL treatment significantly increased cristae abundance in mitochondria that were not associated with a LD (Fig. 1I).

### Electron tomography imaging of cardiac LD-ER associations

**Figure 2:**
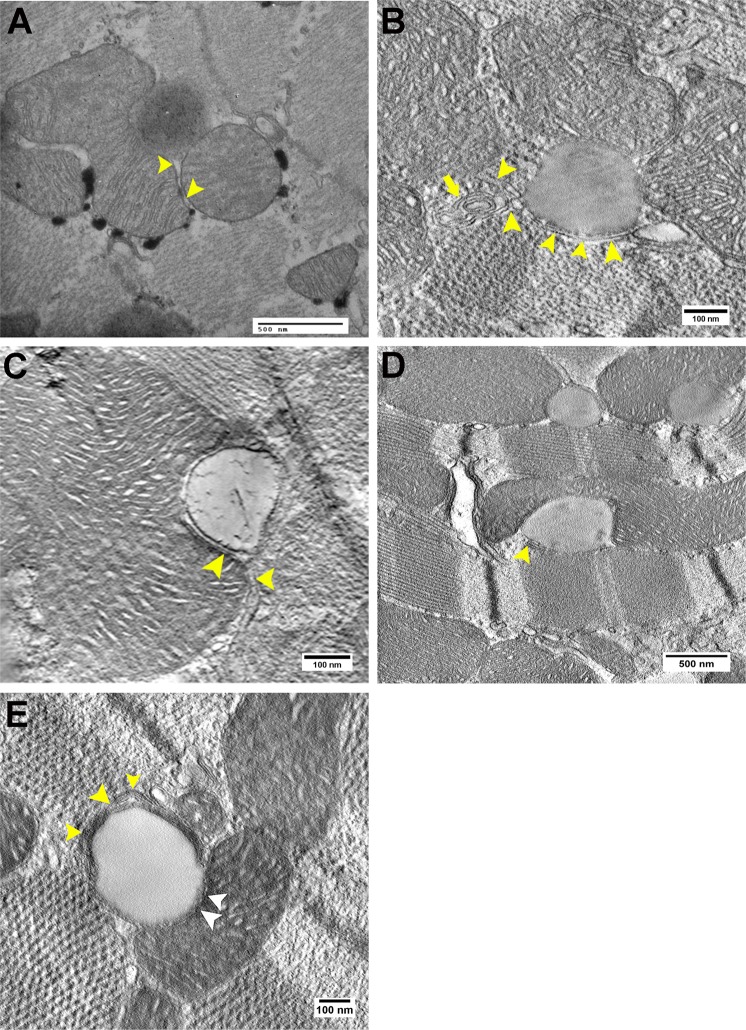
ET imaging of SER-like structures and tightly packed myelin-like figures or lamellar whorls adjacent to LD in cardiomyocytes. (**A**) SER-like channel (arrow heads) directly apposes the LD in a standard TEM micrograph. Scale bar = 500 nm. Representative tomographic slices showing a SER-like channel (arrow head) apposing LD in cardiac tissue obtained from (**B**) Fed, also note peripherally placed lamellar whorls (arrow) circumscribe the myofibrillar aspect of the LD. Scale bar = 100 nm. (**C**) Fasting (24 h) condition. Scale bar = 100 nm and (**D**) CL treated (8 h) conditions. Scale bar = 500 nm. (**E**) Possible occurrence of LD hydrolysis (arrow heads) in cardiac tissue, while stitch-like structures (white arrow heads) are seen on the mitochondrial aspect. Scale bar = 100 nm.

Conventional EM micrographs showed the presence of SER-like channels apposing the cardiac LD and mitochondria (Fig. 2A). However, the continuity between SER-LD could not be determined. Unlike other organelles, the entire ER network is completely continuous at all times, even though it constantly rearranges its structure^60^. Since the ER is a continuous membrane bound organelle, we evaluated both transverse and longitudinal cardiac tissue sections from different conditions in order to study the ER structural association with LDs. In our study, reconstructed tomograms from high-resolution ET studies depicted distinct SER-like structures devoid of ribosomes directly apposing LDs and in continuity with the LD outer membrane in either fed, fasting or CL treated conditions (Fig. 2B–D; Supplementary Movie S2A). Earlier studies in brown fat of young rats showed that in conditions of FA mobilization, myelin-like lamellar structures with a regular pattern of alternating dark and light bands were primarily found in the extracellular space and near LDs of adipocytes^61–63^. The periodicity of the structures, 40 Å, was the same as that of lamellar structures composed of either pure oleic acid or FAs formed by lipolysis of chylomicrons *in vitro*. These studies concluded that lamellar whorls are primarily composed of FAs produced by enzymatic hydrolysis of TAG, in chylomicrons or intracellular LDs. In our study, cardiac tomographic reconstructions also revealed lamellar whorls tightly apposed to LDs and mitochondria (Fig. 2B,E; Supplementary Movie S2B). Lamellar whorls and LD-SER associations in cardiac tissue suggest channeling of lipolytic substrates to and from LD that may designate synthesis and hydrolysis respectively.

**Figure 3:**
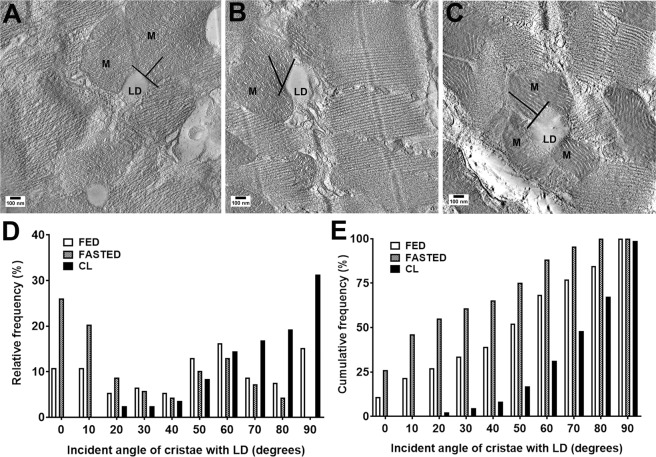
LD size affects mitochondrial cristae orientation. Orientation of cristae relative to the LD-mitochondria contact site quantified on tomogram slices. An incident angle of 0° indicates that cristae lie parallel to the site of contact, whereas an angle of 90° indicates perpendicular cristae. (**A**) fed condition. Scale bar = 100 nm. (**B**) fasting condition. Scale bar = 100 nm. (**C**) CL treated cardiac tissue. Scale bar = 100 nm. (**D**) Relative frequency of cristae angles in fed, fasted and CL treated hearts (skewness; fed = −0.2377, fasted = +0.5265, CL = −1.05. (**E**) Cumulative frequency distribution of cristae angles in fed, fasted and CL treated hearts (χ^2^ value for fed vs fasted = 22.03; **p < 0.05, χ^2^ value for fed vs CL = 75.15; ****p < 0.0001). Note the high distribution of cristae oriented near 0° angles in fasting conditions and near 90° angles in CL treated hearts. N = 25–30 mitochondria/condition; 80–100 cristae segments/condition.

It was recently reported that cristae often orient perpendicularly at inter-mitochondrial junctions, implying functional connectivity^64^. To determine whether the orientation of mitochondrial cristae at the LD-mitochondria interface is influenced by nutritional and pharmacological stimuli, we determined the incident angle of cristae membranes relative to the tangent of adjacent LDs in electron tomograms (Fig. 3). Under fed conditions the majority (~60%) of the cristae exhibited incident angles >50°, with the overall distribution having a skewness of −0.237 (Fig. 3A,D; Supplementary Movie S3A). Fasting significantly decreased the proportion of cristae with high incident angles (>50°) compared to controls (χ^2^ vs Fed = 22.03; **p < 0.05) and the overall skewness of the distribution was +0.265 (Fig. 3B,D; Supplementary Movie S3B). Surprisingly, CL treatment greatly increased the proportion of cristae that were arranged perpendicular or near to perpendicular to the associated LD tangent, with an overall skewness of −1.05 (χ^2^ vs Fed = 75.15; ****p < 0.0001) (Fig. 3C,D; Supplementary Movie S3C). Following CL treatment, ~80% of the cristae had incident angles >50° (Fig. 3E). These observations indicate that the LD-mitochondria interface is dynamic and responsive to metabolic signals. Furthermore, conditions that lead to LD expansion (fasting and CL treatment) can have dramatically different effects on cristae orientation.

### PLIN expression and targeting to the LD-mitochondrial interface with cardiac LD expansion

**Figure 4:**
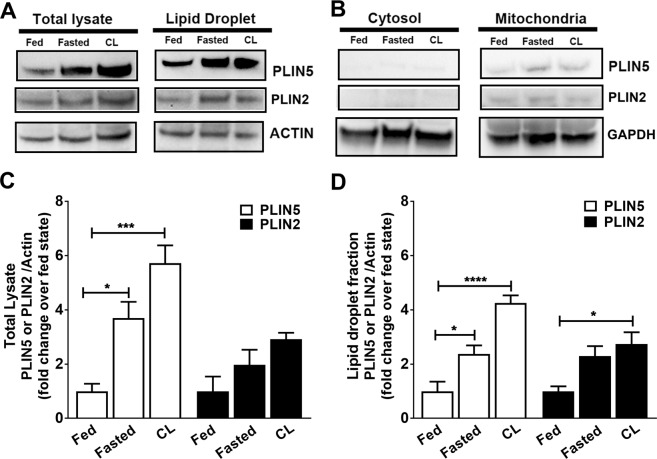
Cardiac LD morphology affects PLIN5 expression at the LD-mitochondrial interface. (**A**) Total lysate, LD, (**B**) cytosol and mitochondrial fractions isolated by density gradient centrifugation of cardiac tissue homogenates from fed, fasted and CL treated conditions. (**C**) PLIN5 and PLIN2 protein levels in total lysates quantified from immunoblots. PLIN5 showed significant increase during fasting and CL activation (*p < 0.05 and ***p < 0.001 respectively, one-way ANOVA). (**D**) PLIN5 and PLIN2 protein levels in LD fractions quantified from immunoblots. PLIN5 showed significant increase during fasting and CL activation (*p < 0.05 and ****p < 0.0001 respectively, one-way ANOVA) over control conditions. PLIN2 protein expression levels also increased under CL activation (*p < 0.05, one-way ANOVA) over control conditions. Both blots were run in parallel. Band intensities were quantified with Image J. The blots were cropped and full length blots are presented in Supplementary Fig. S4.

It is known that fasting increases cardiac expression of PLIN2 and PLIN5 proteins^10,52^. We investigated the expression and subcellular targeting of PLIN2 and PLIN5 during LD expansion induced by fasting and CL treatment. Both fasting and β3-adrenergic activation robustly upregulated PLIN5 protein expression and its targeting to LD fractions (Fig. 4A,C and D). CL treatment also increased expression of PLIN2 compared to fed controls (one way ANOVA, *p < 0.05), whereas the trend to increase levels by fasting was not statistically significant in the LD fractions (Fig. 4A,D). Minor levels of PLIN5 were observed in mitochondrial preparations (Fig. 4B), which likely represents contamination by membranes from tightly associated LD (see below) (Fig. 4B). Cytosolic fractions were nearly devoid of PLIN5 and PLIN2 proteins (Fig. 4B). Full length blots of the cropped images in Fig. 4 are presented in Supplementary Fig. S4.

**Figure 5:**
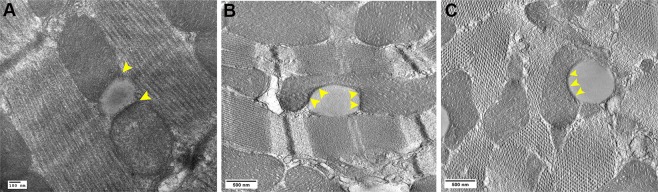
ET imaging of the cardiac LD-mitochondria interface. (**A**) Conventional TEM micrograph shows a LD juxtaposed between two mitochondria within the myofibrillary network. Arrows indicate areas with a ‘stitching pattern’ showing tight apposition of LDs with mitochondria on either side. Scale bar = 100 nm. (**B**) Representative tomographic slice of a 3-D reconstructed longitudinal image obtained by high-resolution electron tomography reveals electron dense regions at the LD-mitochondria interface indicated by arrowheads. Scale bar = 500 nm. (**C**) Cross-sectional tomographic slice also reveals electron dense regions at the LD-mitochondria interface. Scale bar = 500 nm.

Examination of cardiac muscle by TEM showed that intramyocellular LD are aligned between myofibrils and are often punctuated by intervening mitochondria. These mitochondria are closely apposed to LD and a “stitching” pattern was often observed at the interface with standard EM (Fig. 5A). Analysis of high-resolution tomographic slices showed fine structural details missed in conventional micrographs because of overlapping densities. High-resolution ET studies showed electron dense regions in both longitudinal and cross-sectional tomogram slices at the LD-mitochondria interface suggesting potentially different protein densities for LD proteins such as PLIN5 and its binding partners (Fig. 5B,C; Supplementary Movie S5). We further probed these regions with immuno-labeling and electron microscopy studies.

**Figure 6:**
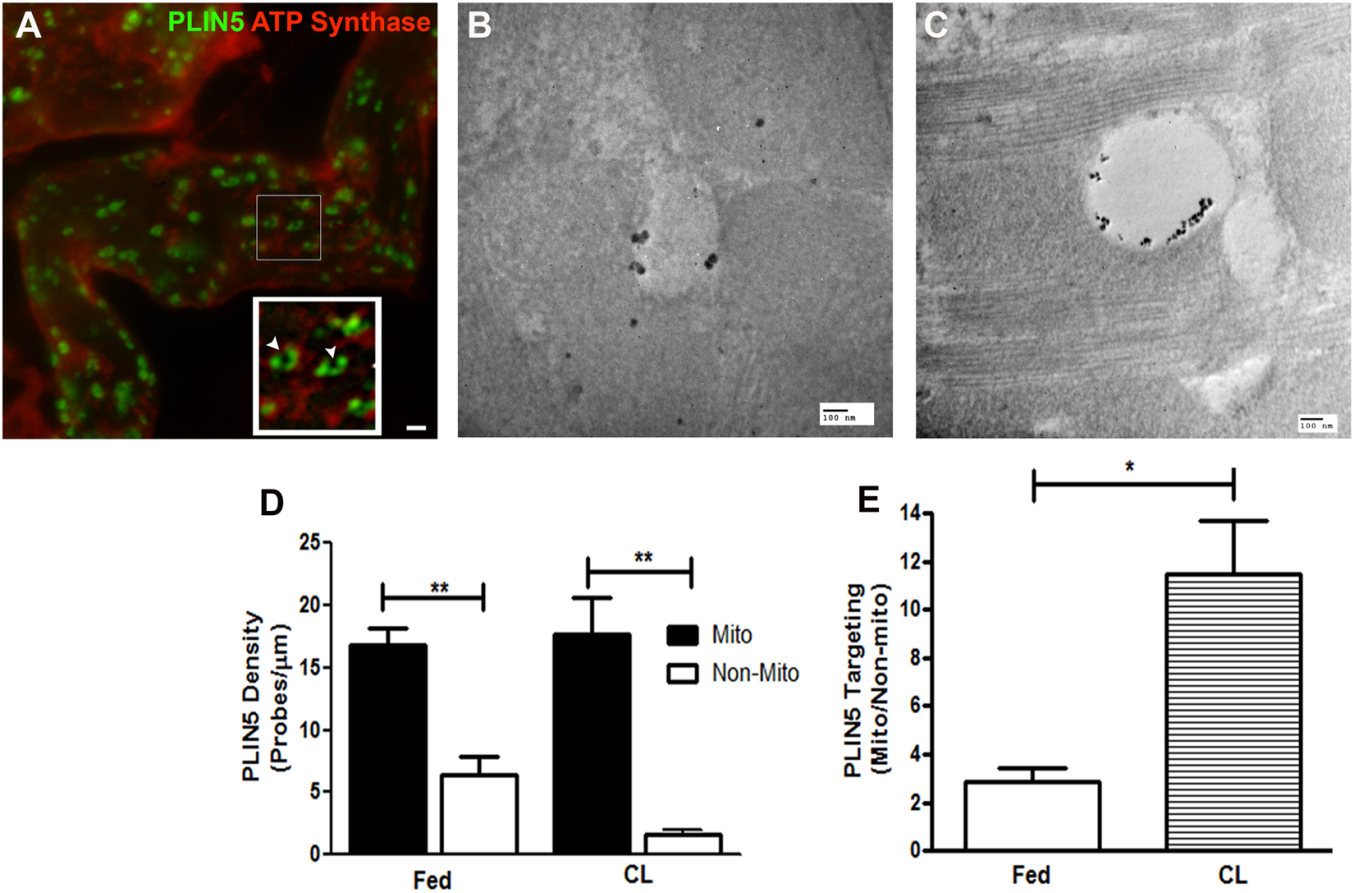
PLIN5 at the junction of LD-mitochondria crosstalk. (**A**) Double-labeled fluorescent image of PLIN5 (Alexa Fluor 488) and ATP synthase (Alexa Fluor 594). Teased cardiac fibers depict PLIN5 localization around the LD. Inset shows enlarged image of individual LDs in the boxed area with ATP synthase in close association with PLIN5. PLIN5 distribution is non-uniform around the lipid droplet (arrowheads) suggesting absence of PLIN5 localization on the non-mitochondrial aspect. Scale bar = 10 µm. (**B**) Cardiac sections from fed condition immunolabeled with PLIN5-FNG shows targeting at the LD-mitochondrial interface. Scale bar = 100 nm. (**C**) Cardiac sections from CL treatment immunolabeled with PLIN5-FNG heavily targeted at the LD-mitochondrial interface. Scale bar = 100 nm. (**D**) Relative abundance of PLIN5 targeting in fed and CL conditions shows that PLIN5 targeting is significantly greater (**p < 0.005, t-test) on the mitochondrial surface vs non-mitochondrial region of LD. (**E**) Density of PLIN5 targeting to the LD-mitochondrial interface is significantly greater in the CL condition (*p < 0.05, t-test).

Histological examination of cardiac sections showed PLIN5 targeted to numerous LDs (Fig. 6A). Confocal imaging of teased cardiac fibers double-labeled for PLIN5 and ATP synthase demonstrated that virtually every individual LD containing PLIN5 immunofluorescence also contained mitochondrial ATP synthase in close proximity. We observed that both ATP synthase (Fig. 6A) and Mitotracker Red labeling (Supplementary Fig. S6A) for mitochondria in whole mount sections were diffused revealing the limitations of resolving the mitochondria at the light level in whole mount cardiac sections. However, the vacancies observed in Fig. 6A surrounded by PLIN5 were confirmed as LDs with LipidTOX (red) labelling in Supplementary Figs S6C and S6D. Interestingly, PLIN5 immunofluorescence did not uniformly surround the LD, but rather showed greatest intensity at the lateral surfaces that face intermyofibrillary mitochondria, suggesting selective targeting to LD-mitochondrial interface (Fig. 6A and Supplementary Fig. S6D). To examine this, we further investigated the localization of PLIN5 protein in myocytes by FNG-labeling in thin section cardiac tissues of basal and CL treated conditions. PLIN5 immunoreactivity was found to be strongly targeted to the interface between the LD and the mitochondria in both fed and CL states. However, no specific labeling of the mitochondrial matrix was observed (Fig. 6B,C). Supplementary Fig. S7A–C show negative controls for immunolabeling of the PLIN5 protein while S7D-F show additional images for PLIN5 labeling in the CL state in the cardiac tissue. Quantification of PLIN5-FNG probe distribution indicated that label was heavily concentrated on LD surfaces that contacted mitochondria vs other LD surfaces in control and CL conditions (Fig. 6D). LD enlargement under CL conditions significantly increased the expression of PLIN5 on the LD-mitochondrial interface (Fig. 6E). We also attempted immunoEM localization of PLIN2; however, although PLIN2-FNG appeared to be targeted to the LD-mitochondrial interface, the labeling was not sufficiently robust for quantitative analysis.

## Discussion

The storage and mobilization of lipid energy are fundamental processes of virtually all mammalian cells. Much of our understanding of mammalian LD is derived from studies of adipocytes where PLIN1 is the major LD scaffold. Much less is known about the characteristics of PLIN proteins in heart or how they might change in response to perturbations that alter both intramyocellular LD content^17^. The purpose of this study was to examine the effects of variations in FA supply on intramyocellular LDs, PLIN proteins and cardiac LD associations with mitochondria and SER.

Our results demonstrate that elevation of adipocyte lipolysis by fasting or acute β3-adrenergic receptor activation elevates myocardial TAG content. We found that fasting increased TAG content by increasing LD size, whereas β3-adrenergic activation increased both LD size and numbers (Table 1). As expected, cardiac LDs were closely associated with mitochondria under basal conditions, and this tight contact was maintained during LD expansion induced by β3-adrenergic receptor activation. Interestingly, CL treatment increased cristae abundance in mitochondria that were distant from LDs, suggesting that these mitochondria might accommodate excessive FA supply with elevated oxidative capacity.

**Table 1:** Summary of the effects of fasting and CL- stimulated lipolysis in cardiac tissue.

	LD number	LD size	Mitochondrial cristae abundance	Mitochondrial cristae angles at LD-mitochondria interface	PLIN5 protein in LD fraction	PLIN2 protein in LD fraction	PLIN5 targeting to LD-mitochondrial interface
Fasted	< >	+++	< >	<50° angle	++	< >	−
CL treated	+++	+++	+++	>50° angle	+++	++	+++

The summary table depicts the responses of LD, mitochondria and LD-associated proteins in cardiac tissues when lipolysis is stimulated with fasting or CL. < > no significant change, − no data.

Recent work has shown that mitochondria participate in inter-organelle communication that can involve structural associations, including intermitochondrial junctions and mitochondrial nanotubes. Interestingly, cristae have been shown to align at intermitochondrial junctions, suggesting coordination and communication across these organelles^64^. We observed that electron tomography - the three-dimensional high-resolution imaging of thick tissue samples was critical to provide evidence of cristae orientation through the thickness of the mitochondria. We found that the alignment of cristae of intermyofibrillary (IMF) mitochondria that appose LD is responsive to nutritional and pharmacological challenges. Under basal conditions, most cristae of IMF mitochondria aligned perpendicular to the LD surface. Interestingly, although both fasting and CL treatment increases LD size and TAG content, these treatments had dramatically different effects on cristae orientation. Whereas fasting decreased the perpendicular orientation, β3-adrenergic activation greatly increased the fraction of cristae that were oriented perpendicularly to the LD. These results suggest that the mitochondrial contact site and cristae organizing system (MICOS) that connects the inner and outer mitochondrial membranes might coordinate with LD protein complexes (like those containing PLIN5). In this regard, recent work in brown adipocytes^65^ shows that mitochondria associate with LD under conditions that favor FA esterification and dissociate under conditions of FA mobilization and oxidation. In this study, mitochondria bound to LDs, known as peridroplet-mitochondria (PDM), were oriented perpendicular to the bound LD, had increased pyruvate oxidation, electron transport, and ATP synthesis capacities, but reduced fusion-fission activities^65^. In our study, CL treatment provoked massive release of FA from adipose tissue and triggered pronounced release of insulin, which facilitates FA uptake and re-esterification^56,66,67^. It is thus possible that CL treatment and fasting have opposite effects as to whether FA are sequestered or oxidized. Although the molecular basis of LD-cristae orientation is currently unknown, these observations nonetheless clearly illustrate that the interface between mitochondria and LD is sensitive to metabolic signals. We note that IMF mitochondria in the heart are largely immobile^68^, and interactions with MICOS would be one means of rapidly regulating functional interactions. The relation between cristae shape and the OXPHOS system is multifaceted and many mitochondrial-shaping proteins are coordinately involved. Proteins such as optic atrophy 1 (OPA-1), myeloid cell leukemia 1 (MCL-1), prohibitin (PHB), stomatin-like protein 2 (SLP2), and ATP synthase have been implicated in maintaining cristae morphology and function^69,70^.

High-resolution ET studies probing the channeling of FAs in the cardiac tissue showed distinct features suggesting the trafficking of lipolytic products in cardiac tissue. Previous work by Blanchette-Mackie and others showed that lamellar structures develop in adipose tissue under conditions causing lipolysis, accumulation of FA in tissue and partial ionization of FA^61,62,71^. Tomographic reconstructions provide evidence of lamellar whorls apposing LD and mitochondria in the cardiac tissue. The presence of such lamellar structures around LDs in cardiac tissue may indicate possible FA movement for β-oxidation. The tightly packed peripheral whorls around LDs in the CL-treated group implicate a role for PLIN5 in regulating TAG hydrolysis and/or FA mobilization. SER-like structures were also found in close proximity to the lamellar whorls. Evidence of SER structures traversing the space between adjacent mitochondria or closely wrapping around LDs were found in tomographic reconstructions. Furthermore, ET imaging revealed membrane continuity between smooth ER and LD membranes. The significance of these associations is not completely understood but could indicate that intramyocellular LDs are interconnected for possible FA trafficking.

Confocal immunofluorescence studies suggested that PLIN5 was concentrated on LD surfaces that appose mitochondria, while electron microscopic analysis demonstrated that the PLIN5-FNG was indeed highly targeted to the cardiac LD-mitochondria interface (Table 1). Previous investigators reported that PLIN5 is targeted to mitochondrial inner membrane^72^. However, the present data and recent results by Wang and others^73,74^ indicate that PLIN5 exclusively targeted to LD or the LD/mitochondria interface. Importantly, precise targeting of PLIN5 to the mitochondrial surface was maintained as LDs grew in response to β3-adrenergic receptor activation or fasting. Changes in PLIN5 expression with excessive FA supply imply that PLIN5 exerts a tight control over the FA flux into mitochondria for β-oxidation. Our data adds to the growing evidence that PLIN5 might have a significant role in coupling LD-FA release to mitochondrial FA oxidation^10,19^.

The interface between LD and mitochondria is enriched in a special subset of proteins. PLIN5, which is highly targeted to the LD surface apposing mitochondria, separately binds ATGL, the rate-limiting step for lipolysis, and its activating protein, ABHD5^33,73–76^. We recently reported that long-chain acyl-CoA binds ABHD5 and promotes its association with PLIN5^77^. Therefore, upregulation of PLIN5 protein at the LD-mitochondrial interface would suppress lipolysis by sequestering ABHD5 and preventing activation of ATGL in times of excess FA supply. In summary, the present results demonstrate the dynamic regulation of cardiac LD size, number, and mitochondrial association in response variations in systemic FA supply. Furthermore, immuno-EM and ET results strongly support the existence of a specialized and dynamic ‘metabolic synapse’ between cardiac LD and mitochondria that provides local control of FA storage, mobilization and oxidation.

## Materials and Methods

### Ethics statement

All experiments were performed according to the protocols reviewed and approved by the Institutional Animal Care and Use Committee and the Division of Laboratory Animal Resources (DLAR) of Wayne State University. All efforts were made to minimize animal discomfort.

### Physiological and pharmacological treatments

Seven to eight week-old C57BL/6J mice (Jackson Laboratory, Bar Harbor, ME) were used for all experiments. Mice were either *ad libitum* control, fasted for 24 h, or injected intraperitoneally with CL316,243 (CL; 10 nmol), a β3 adrenoceptor agonist, 8 h prior to sacrifice. The mice were anesthetized with an intraperitoneal injection of Avertin (Sigma-Aldrich) prior to perfusion for electron microscopy tissue collection.

### Fluorescence microscopy

For double labeling of PLIN5 and ATP synthase, cardiac tissue sections were incubated with affinity-purified rabbit anti-PLIN5 (1:500)^76^ overnight at 4 °C, and mouse anti-ATP synthase (1:500; Abcam) for 2 h at room temperature, and detected with secondary Alexa Fluor 488 and 594 conjugated antibodies (Molecular Probes). Labeling was also performed with Mitotracker red CMX (Cell Signaling) for mitochondria and LipidTOX Deep Red (Invitrogen) for LDs in cardiac tissue sections. Images were acquired with an Olympus IX-81 microscope equipped with automated filter controls and a spinning disc confocal unit. Images were captured using a 60 X 1.2 NA plan apo water immersion lens and a Hamamatsu ORCA cooled CCD camera. Microscope control and data acquisition were performed using IP labs (Scanalytics, BD Biosciences) software.

### Transmission electron microscopy

Immediately after anesthesia, mice hearts were perfused with 4% paraformaldehyde in PBS for about 5 mins. Hearts were excised and ventricles were cut into 1 mm^2^ pieces in the perfusion fixative. Tissue samples were fixed overnight at 4 °C in 4% paraformaldehyde, 1.25% glutaraldehyde, 0.2% sucrose and 25 mM HEPES in 0.12 M sodium cacodylate buffer. Secondary fixation was performed with 3% glutaraldehyde and 4% tannic acid in cacodylate buffer for 1 h at 37 °C. Tissues were post-fixed with 2% osmium tetroxide in cacodylate buffer for 2 h at 4 °C followed by dehydration in grades of ethanol, and then treated in 100% propylene oxide. The tissues were then treated with 100% acetone and embedded in Durcupan ACM resin or EMBed resin (Electron Microscopy Sciences) and polymerized. Thick sections (300–500 nm) were cut from intact, Durcupan-embedded samples using a RMC microtome (Boeckler Instruments; Tucson, AZ)) and collected onto clamshell grids. Thin (70–90 nm) sections were cut from EMBed resin blocks and collected onto formvar-coated or uncoated copper grids. They were post-stained with 2% aqueous uranyl acetate and Reynold’s lead citrate. Thin sections were surveyed on a conventional intermediate voltage (200 kV) TEM (JEOL JEM-2010; JEOL Ltd.). Three animals per group were used, and more than 10 positions per sample were studied.

### Fluoronanogold immuno-electron microscopy

Ultra-thin LR White resin embedded cardiac tissue sections were immuno-labelled with affinity purified rabbit anti-PLIN5^76^ or guinea pig anti-PLIN2 (Fitzgerald, MA) antibodies. Primary antibodies were detected with anti-rabbit Alexa Fluor 594 FNG or anti-guinea pig Alexa Fluor 594 FNG (Nanoprobes). Nanogold particles were silver enhanced to visualize the gold particles at the electron microscopic level^78^. Sections were contrasted with uranyl acetate and lead citrate for TEM imaging.

### Electron tomography

Tomographic studies of Durcupan resin embedded cardiac tissue samples were conducted at National Center for Microscopy Imaging and Research (NCMIR), University of California, San Diego. 10 or 20 nm colloidal gold particles were added to the sections prior to imaging. The gold particles acted as fiducials for alignment of the tilt series. Raw tilt images were captured using a Tecnai Titan TEM (FEI instruments; Hillsboro, OR) at 300 kV and a JEOL 4000 TEM (Tokyo, Japan) at 400 kV, equipped with SerialEM (University of Colorado; Boulder, CO) software and a TEMCam F-2242K CCD camera (TVIPS; Gauting, Germany). Prior to data acquisition, the sections were pre-irradiated in the electron beam at low magnification/low beam current at 0° and ±60° for 10 min. Tilt series consisting of 121 images taken at one degree increments over a range of −60 to +60 degrees were produced. Tomogram alignments and weighted back-projection reconstructions were performed using IMOD VR4.3 software (Boulder Laboratories for 3D Electron Microscopy of the Cell, University of Colorado). Finally, movies were created in IMOD from the reconstructions.

### Tissue fractionation and Western blot

Freshly excised mouse hearts were homogenized in RIPA buffer (Teknova Hollister, CA) containing 150 mM sodium chloride, 1% Triton X-100,1% deoxycholic acid-sodium salt, 0.1% sodium dodecyl sulfate, 50 mM Tris-HCL (pH 7.5), 2 mM EDTA and protease inhibitor as directed by the manufacturer (Roche Diagnostics). For tissue fractionation, heart tissues were minced, homogenized in 20 mM HEPES (pH 7.5), 1 mM EDTA and 20% sucrose buffer containing protease inhibitors. Post-nuclear supernatants were centrifuged at 15,000 × g for 15 min at 4 °C in a fixed angle rotor to obtain mitochondrial pellet and a crude LD fraction. LD fractions were overlaid with HEPES buffer containing 10% and 0% sucrose and then centrifuged at 100,000 × g for 30 min at 4 °C in a Beckman TL 100 Ultracentrifuge (Beckman Coulter, CA). The centrifuge tubes were then frozen, and the LD fractions were obtained by scraping the top 2 mm layer of the tubes. Proteins in the LD fractions were precipitated with acetone. LDs were obtained following two rounds of gradient centrifugation. Protein concentrations were quantified and the samples were immunoblotted^79^. Blots were probed with affinity-purified rabbit anti-PLIN5^76^, affinity-purified rabbit anti-PLIN1, guinea pig anti-PLIN2 (Fitzgerald, MA). Primary antibodies were detected with donkey anti-rabbit horse-radish peroxidase, goat anti-guinea pig horse-radish peroxidase (Cell Signaling Technology) and Supersignal chemiluminescence reagents (Pierce, Rockford, IL). Actin (goat, Santa Cruz) and GAPDH (rabbit, Santa Cruz) were used as loading controls.

### Triglyceride and fatty acid assay

Cardiac tissues (100 mg) were excised, weighed, and minced. Tissues were homogenized in 1 ml of 5% NP-40/ddH_2_O solution using a Dounce homogenizer or pestle with 10–15 passes. Total lipids were extracted by slowly heating the samples to 80–100 °C in water bath for 2 to 5 minutes or until the NP-40 became cloudy, then cooled down to room temperature. The heating step was repeated one more time to solubilize all triglyceride. Samples were then centrifuged for 2 minutes at top speed using a micro-centrifuge to remove any insoluble material. TAG content in the supernatant was determined enzymatically (TR0100, Sigma-Aldrich) according to the manufacturer’s protocol. Fatty acids were determined with the NEFA reagent (WAKO diagnostics).

### Image analysis

LD number, size, LD-mitochondria contact area measurements were performed on electron micrographs of cardiac sections with Image J (NIH, Bethesda). More than five different grids from three to four different control and experimental group mice were studied. LD numbers were counted manually. PLIN5-FNG and PLIN2-FNG probe densities were determined manually from thin section electron micrographs. The cristae abundance was calculated from the ratio of crista/outer mitochondrial membrane (OMM) perimeters. Measurements of cristae abundance were determined with reconstructed tomograms and Image J. This ratio was interpreted as the ATP synthesizing capacity of the mitochondrion^59^. Perimeters of mitochondria in close association with LDs and the perimeters of total cristae within the mitochondria were determined from reconstructed tomographic slices with Image J. Discrete membrane associations with LDs in cardiac thick sections (300–500 nm) were examined with ET. Mitochondrial cristae orientations relative to the LD contact site were measured in electron tomograms with ImageJ. Several consecutive slices within each of the reconstructed tomogram were analyzed to determine the cristae orientations in the mitochondria. For each condition, a representative reconstructed tomogram with several slices merged together as a movie is provided in the Supplementary Information. In total, 7–8 reconstructed tomograms per condition showing two to three mitochondria with one or more LDs in each tomogram slice were analyzed. If cristae were perfectly perpendicular to the LD contact site, the angle resulted in a 90**°** angle, whereas cristae membranes parallel to the LD-mitochondria contact site resulted in a 0° incident angle.

### Statistical analysis

All statistical analyses were conducted with Graph Pad Prism software version 5. Data are presented as mean ± SEM. Statistical significance between two groups was determined by unpaired t-test, one-way or two-way ANOVA. Differences with *P* value of <0.05 were considered statistically significant. Chi square tests were performed with 95% confidence interval.

## Supplementary information

Supplementary Movie S2A

Supplementary Movie S2B

Supplementary Movie S3A

Supplementary Movie S3B

Supplementary Movie S3C

Supplementary Movie S5

Supplementary figure legends and figures
