# Supplementary material for: Adipocyte lipolysis affects Perilipin 5 and cristae organization at the cardiac lipid droplet-mitochondrial interface
Source: Sci Rep. 2019 Mar 18;9:4734. doi: 10.1038/s41598-019-41329-4 (PMC6426865; doi:10.1038/s41598-019-41329-4)
Supplement: Supplementary file 7 — Supplementary figure legends and figures [file 41598_2019_41329_MOESM7_ESM.pdf]

Adipocyte lipolysis affects Perilipin 5 and cristae organization at the cardiac lipid droplet-mitochondrial interface

Author information:

Mita Varghese [1], Victoria A. Kimler [1], Fariha R. Ghazi [1], Gurnoor K. Rathore [1], Guy A. Perkins [2], Mark H. Ellisman [2] and James G. Granneman [1].

[1]Center for Integrative Metabolic and Endocrine Research, Wayne State University School of Medicine, Detroit, MI 48201, USA.
[2]National Center for Microscopy and Imaging Research, University of California, San Diego, La Jolla, CA 92093, USA.

Supplementary Figure S1. Serum FFA levels in *ad libitum* fed, fasted and CL treated conditions. N= 5 mice/condition; ***$p<0.0001$.

Supplementary Movie S2A. Tomogram reconstruction depicting ER channel in continuity with cardiac LD.

Supplementary Movie S2B. Tomogram reconstruction depicting lamellar whorls in apposition with cardiac LD.

Supplementary Movie S3A. Cristae orientation of mitochondria associated with cardiac LD in the fed *ad libitum* state. Movie represents consecutive tomogram slices merged to depict some cristae orientation near 90° angles.

Supplementary Movie S3B. Cristae orientation of mitochondria associated with cardiac LD in the fasted state. Movie represents consecutive tomogram slices merged to depict cristae orientation less than 90° angles or parallel to LD.

Supplementary Movie S3C. Cristae orientation of mitochondria associated with cardiac LD in CL treated state. Movie represents consecutive tomogram slices merged to depict cristae orientation near 90° angles.

Supplementary Figure S4. Full length blots of proteins in cardiac total lysate, lipid droplet, cytosol and mitochondria fractions (shown as cropped images in Figure 4). PLIN5 (top), PLIN2 (middle) and beta-actin or GAPDH (bottom) in **A.** Total and LD fraction **B.** cytosol and mitochondrial fraction. The dark band in (B) the PLIN2 blot for cytosol and mitochondria fraction is un-stripped GAPDH.

Supplementary Movie S5. Electron dense regions at the LD-mitochondrial interface. The LD-mitochondrial interface depicts an electron dense region with stitching pattern.

Supplementary Figure S6. Immunofluorescence imaging of LD-mitochondrial interface. Micro-dissected cardiac tissue depicting labeling for **A.** Mitochondria (Mitotracker red CMX). **B.** PLIN5 (Alexa Fluor 488). **C.** LD (LipidTOX Deep Red). Scale bar = 10µm. **D.** Inset shows enlarged and merged image of individual LDs with PLIN5 (LipidTOX-PLIN5) in the boxed areas in B and C. PLIN5 immunofluorescence is not symmetric and appears to be more intense along the axis of the myofibril.

Supplementary Figure S7. PLIN5 FNG labeling in cardiac tissue. **A-C.** Negative control (no primary antibody) for PLIN5-FNG labeling depicts non-specific labeling. Scale bar = 500 nm. **D-F**. PLIN5-FNG labeling in CL treated cardiac tissue depicts specific labeling on LD-mitochondria interface. Scale bar =100 nm and 500nm.

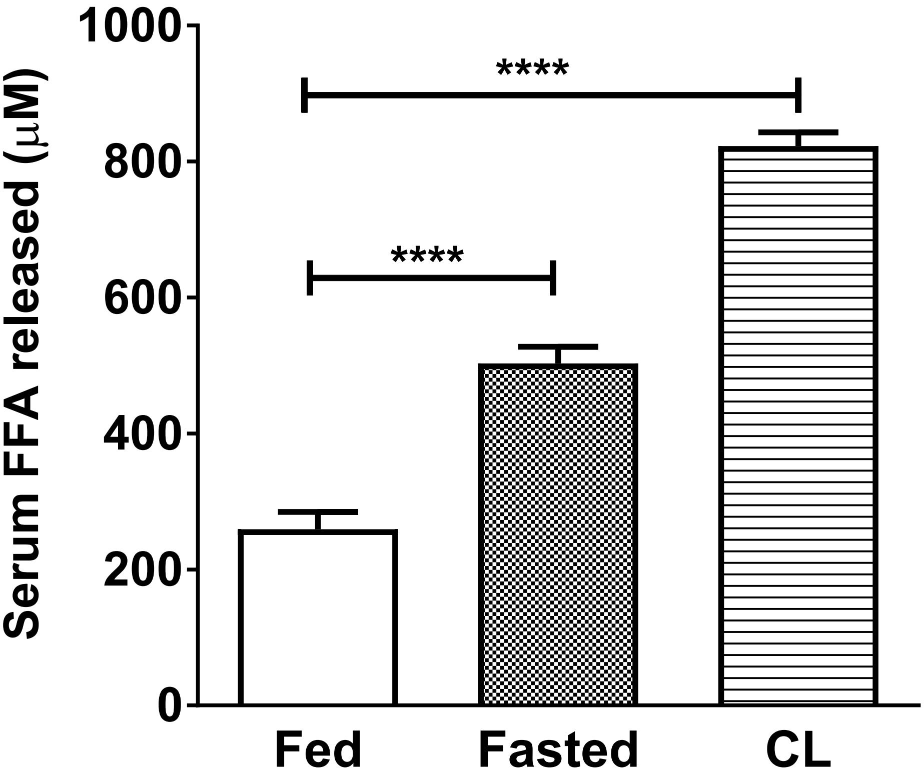

Supplementary Figure S1. Serum FFA levels in *ad libitum* fed, fasted and CL treated conditions.

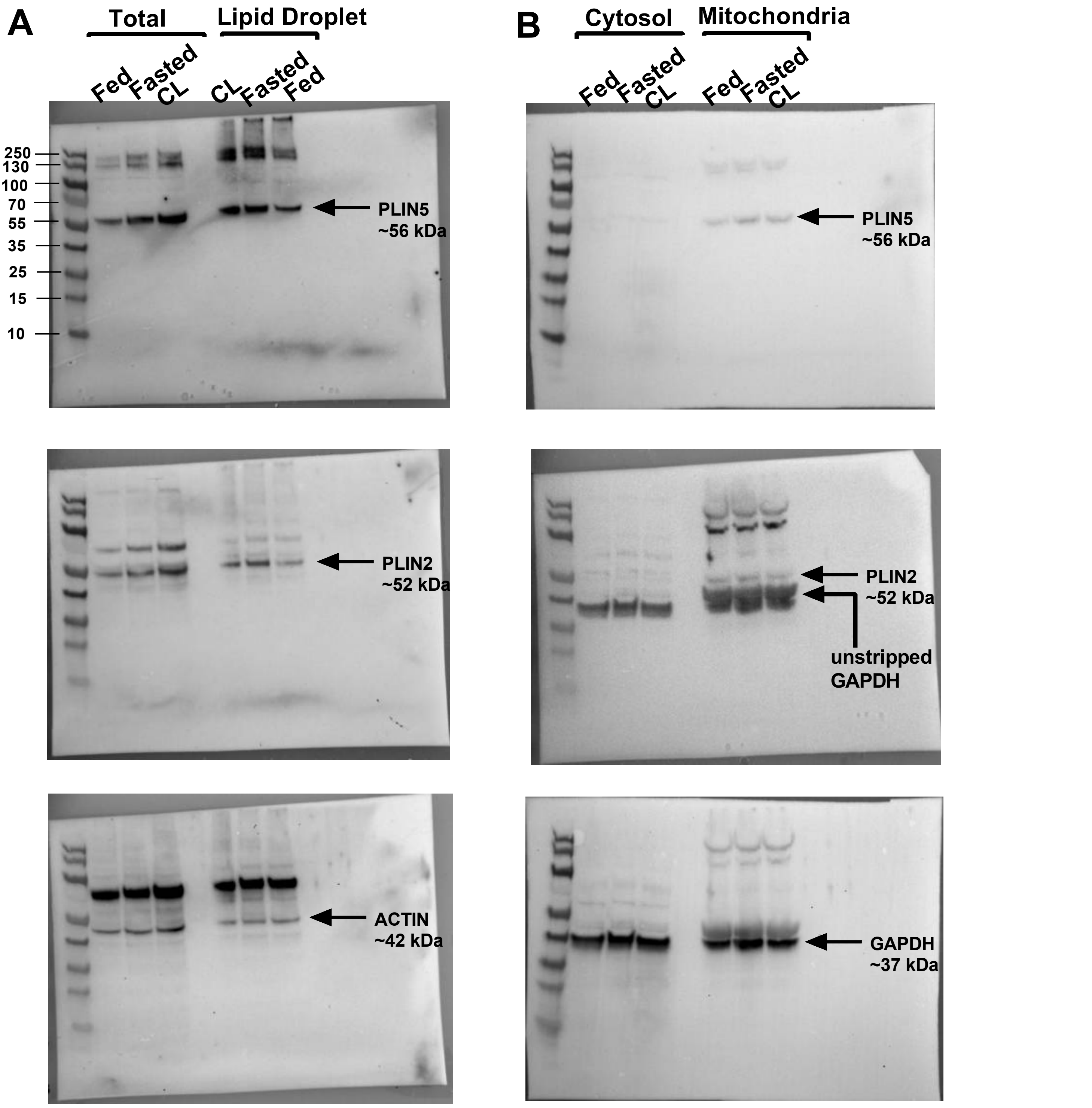

Supplementary Figure S4. Full length blots of proteins in cardiac total lysate, lipid droplet, cytosol and mitochondria fractions (shown as cropped images in Figure 4).

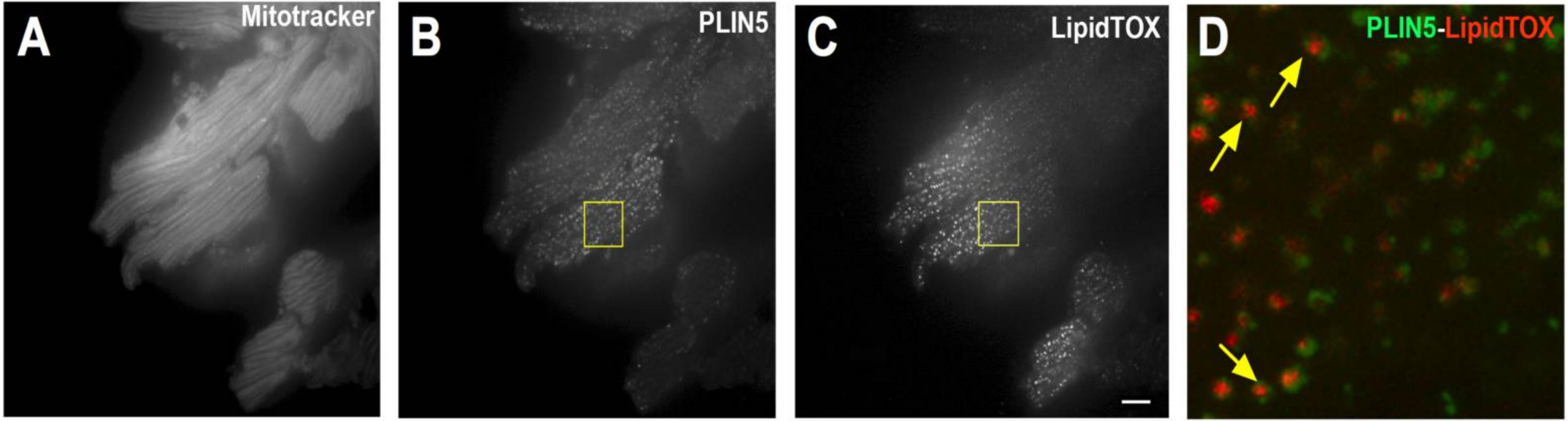

Supplementary Figure S6. Immunofluorescence imaging of LD-mitochondrial interface.

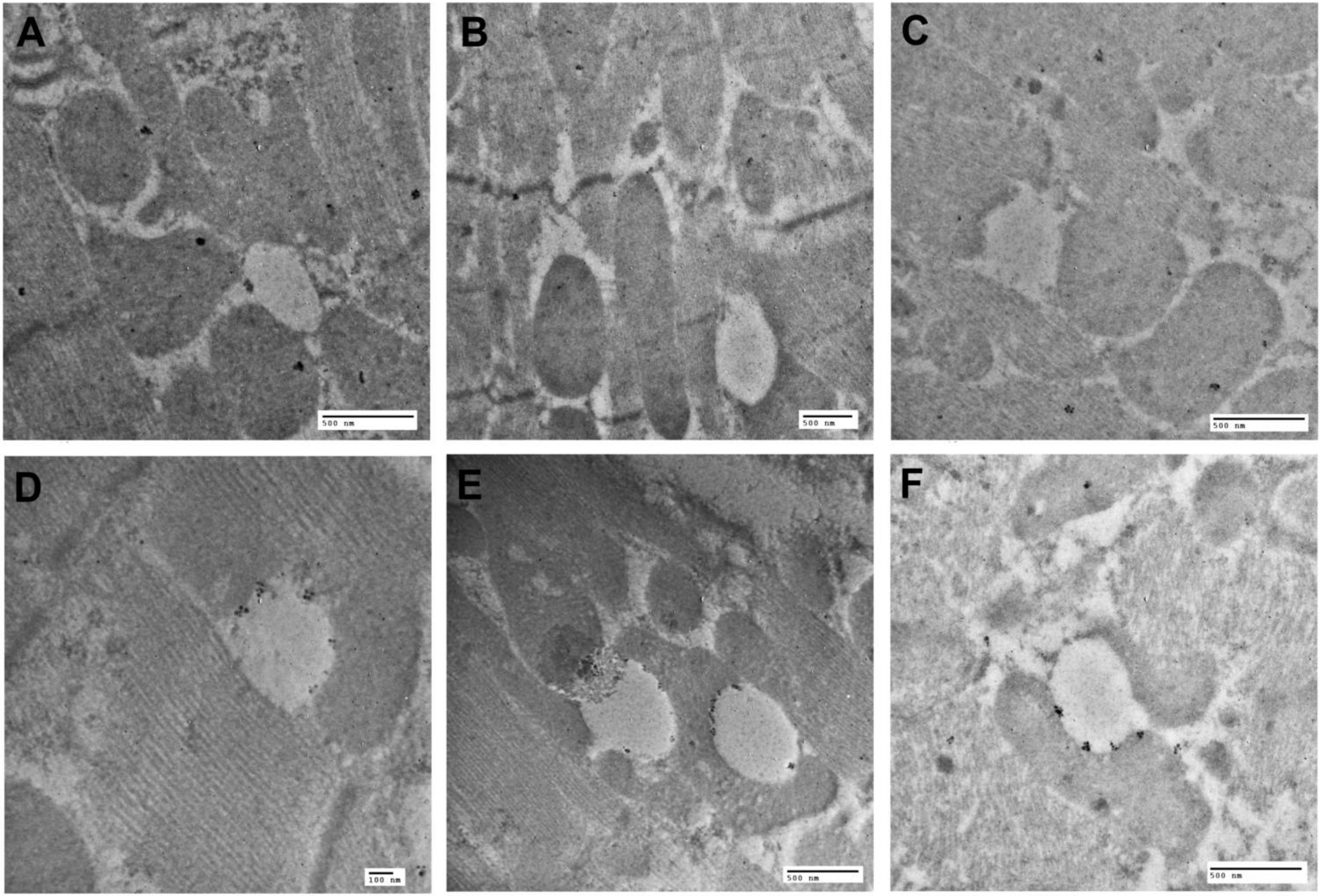

Supplementary Figure S7. PLIN5 FNG labeling in cardiac tissue.
